# Obstacle Detection System for Agricultural Mobile Robot Application Using RGB-D Cameras

**DOI:** 10.3390/s21165292

**Published:** 2021-08-05

**Authors:** Magda Skoczeń, Marcin Ochman, Krystian Spyra, Maciej Nikodem, Damian Krata, Marcin Panek, Andrzej Pawłowski

**Affiliations:** 1 Unitem, ul. Kominiarska 42C, 51-180 Wrocław, Poland; marcin.ochman@unitem.pl (M.O.); krystian.spyra@unitem.pl (K.S.); maciej.nikodem@pwr.edu.pl (M.N.); damian.krata@unitem.pl (D.K.); marcin.panek@unitem.pl (M.P.); andrzej.pawlowski@unitem.pl (A.P.); 2 Faculty of Electronics, Wrocław University of Science and Technology, Wybrzeże Wyspiańskiego 27, 50-370 Wrocław, Poland

**Keywords:** RGB-D camera, vision pipeline, obstacle detection, obstacle mapping, mapping accuracy, autonomous lawn mower

## Abstract

Mobile robots designed for agricultural tasks need to deal with challenging outdoor unstructured environments that usually have dynamic and static obstacles. This assumption significantly limits the number of mapping, path planning, and navigation algorithms to be used in this application. As a representative case, the autonomous lawn mowing robot considered in this work is required to determine the working area and to detect obstacles simultaneously, which is a key feature for its working efficiency and safety. In this context, RGB-D cameras are the optimal solution, providing a scene image including depth data with a compromise between precision and sensor cost. For this reason, the obstacle detection effectiveness and precision depend significantly on the sensors used, and the information processing approach has an impact on the avoidance performance. The study presented in this work aims to determine the obstacle mapping accuracy considering both hardware- and information processing-related uncertainties. The proposed evaluation is based on artificial and real data to compute the accuracy-related performance metrics. The results show that the proposed image and depth data processing pipeline introduces an additional distortion of 38 cm.

## 1. Introduction

In complex robotic tasks, such as in agricultural applications, sensing systems are a key element influencing the functionality and effectiveness of job execution. Proper selection of the sensing system is usually made in the light of defined functionalities and requirements established at the robot design stage. In this context, a sensory system based on computer vision has a special place due to its versatility. It can be used in numerous applications, ranging from fruit detection (including size, color, ripeness, etc.) to object tracking and establishing the robot path after scene analysis [1]. For this reason, vision systems are the basis for autonomous robot navigation, since in a large number of applications, paths are marked/defined/detected visually [2]. As an example, the orchard’s robot navigation can be introduced, where the mobile robot needs to detect the path autonomously (e.g., defined as the position in the middle of the row) to accomplish their task (recollection, spraying, crops stand counting, plant phenotyping, etc.) [3,4,5,6]. In agricultural applications, the path detection and navigation strongly depend on the robotic task goals. There are different ways the path is specified/defined, and various constraints affect robot operations, including image recognition and maneuvering—the most typical examples include road detection [7], crop row guidance for agriculture robots [3,8], area detection for lawn movers [9], and obstacle detection and procedures for their avoidance [10].

Simultaneously with the task’s objectives, most autonomous robots need to detect and avoid obstacles to ensure safe operation. Obstacle detection systems can include a large number of sensors and techniques ranging from ultrasonic sensors and lidars to more complex solutions relying on computer vision systems. Due to their flexibility, cameras are universal and widely applicable in tasks where a complex perception system is required [1,5,6]. In this context, physical camera characteristics, such as resolution, field of view, etc., are only part of the overall computer vision system properties. In these systems, most features are defined by the implemented software and data processing algorithm. For this reason, in robotic applications, where the computer vision system is used for navigation purposes (e.g., obstacle detection and mapping), the accuracy error introduced by the processing software should also be considered to improve the localization and navigation effectiveness. The robotic agricultural application considered in this work, lawn mowing, assumes the robot knows only the working area boundary, expressed by the GPS coordinates, and the robot’s main objective is to cover the working area [9,11]. To complete this goal, the robot global path is generated to optimize robot movements and to perform tasks more efficiently. However, in this approach, the robot has no information regarding the obstacles’ positions and needs to detect and avoid them in an autonomous manner using its sensors. Detected obstacles need to be mapped in order to be avoided by the robot. To accomplish this task, the robot’s global path must be divided into smaller parts (e.g., using the idea of moving horizon), known as a local plan. Afterwards, the local planner takes into account the global goals and obstacles and generates the local plan, allowing obstacles to be avoided and simultaneously performing as close as possible to the global path. The described approach is widely used in many agricultural tasks [12,13]. The performance of the job execution depends mainly on the type (static or dynamic) and the number of obstacles, influencing the obstacle mapping precision and, as a consequence, obstacle avoidance performance and effective area coverage. Additionally, for safety reasons, the obstacle’s shape is augmented (“inflated”) with a safety distance that is added to the original dimension to make the avoidance procedure safe [10,14]. To make this navigation strategy efficient (maximize area coverage), obstacles in working areas should be localized with high precision. In practical applications, obstacle localization is usually not very precise, the safety distance is overestimated (to promote safety over performance), and the efficiency in area coverage drops. However, if one can improve the estimation of the required safety distance, the criteria for both safety and performance can be satisfied, while the area coverage can be maximized. This paper describes a novel methodology to determine the obstacle detection accuracy for a RGB-D camera-based vision system. The main objective is to compute the real precision of the system considering both hardware-related and information processing pipeline (software)-related accuracy. The methodology used here exploits virtual and real data to calculate the accuracy error related to the sensor and to the data processing pipeline. In this approach, artificial data are used to determine the average mapping distortion introduced by the software processing. Using this information and computed indexes from the real scene analysis, the accuracy related to the sensor precision can be established. As a consequence, the real accuracy of obstacle mapping is determined, and then the real precision can be considered in the planning algorithm for obstacle avoidance. Thanks to this, the safety margin can be set within real needs, resulting in better planning and an improved ratio between working and covered areas.

The main contribution of the paper is related to a new evaluation method for obstacle detection, i.e., marking obstacles on the map, which is used by the navigation module. To determine the mapping accuracy, a new metric in terms of obstacle detection was defined. The described research has also shown that segmentation metrics are not sufficient for vision/depth systems in terms of obstacle detection and the navigation module, and additional metrics are required to unequivocally determine the mapping performance. The proposed accuracy verification method was tested on sequences collected from real sensors. The experimental evaluation performed confirmed the effectiveness of the introduced method.

This paper is organized as follows: Section 2 presents a detailed analysis of related works. Section 3 summarizes the system architecture used for obstacle detection. In the first place, vision based techniques for obstacle detection are described. Subsequently, it is shown how these obstacles are mapped and handled in a navigation task. The motivation and methodology for accuracy determination in the presented system are also proposed. Section 4 deals with the experimental evaluation of the proposed methodology using artificial and real data sets. Additionally, the verification measures and the procedure for the avoidance system setup, including determined accuracy, are proposed. Finally, conclusions are given in Section 5.

## 2. Related Work

Movement, navigation, and obstacle avoidance are crucial aspects of all robotic systems, irrespective of robot design, application field, and performed tasks (e.g., [15,16,17].) While most agricultural robots move on fields and navigate their path along crop or orchard rows [3,8,15,18], lawn mowers traverse soft surfaces, require high maneuverability, and navigate through an area which is quite uniform with no natural landmarks to follow. This makes it more challenging to navigate the robot, detect deviations from the desired path, and complicate obstacle detection. As a result, the safe operation of lawn mowers requires various sensing technologies including RGB and depth cameras [19,20].

### 2.1. Obstacle Detection

Ball et al. [15] developed a vision-based obstacle detection and navigation for a robot operating in the crop field. The robot navigates the area using a fusion of GPS, inertial sensors, and a camera sensor to follow the crop rows. An additional pair of stereoscopic wide angle cameras are used to observe the area 10 m ahead of the robot and detect obstacles. Obstacles are detected by looking for so-called novelty in the cropping image [21]. The method aims to detect the difference between the current and recent image-detecting candidates for obstacles. This is possible for robots operating in fields where crops/stubble are straight and parallel because the majority of crops and stubble can be removed from consideration, simplifying image processing. Unfortunately, this approach generates a number of false positive detections, especially when the robot moves across the crop rows and turns at the headland turning regions. Additionally, it is sometimes difficult to distinguish between obstacles and the ground when the obstacle is close to the ground. Delayed detection and a short range make it challenging for the robot to respond to the detected obstacles correctly.

Fleischmann et al. [22] also developed a system based on stereo vision. The whole classification algorithm is based on splitting the generated point cloud into cells and the further analysis of the cells, including the density and distribution of the points and neighborhood of the cell.

The use of an RGB camera and distance measurement was investigated by Brown et al. [23]. They attempted to use a monocular vision camera and a single range measurement to improve depth estimation in agriculture settings. The goal was to mitigate the ambiguity of the scale that exists when only vision images are considered. The results presented show improvements when run on NYUv2 and KITTI datasets but fail to work correctly on agricultural objects which exhibit fractal properties.

Past articles [19,20] present a platform for the evaluation of human detection in autonomous mowers using RGB, stereoscopic, infrared and 360° cameras, LIDAR, and radar. The result of these works is the performance analysis for human detection in different settings and poses. Additionally, they developed a database of sensor recordings and corresponding annotations that can be used for the evaluation of other obstacle detection algorithms. The database includes recordings and locations of humans, buildings, vehicles, and vegetation.

Christiansen et al. [17] proposed a method for obstacle perception using anomaly detection. They benefit from combining deep convolutional neural network (CNN) with anomaly detection to solve the problem of detecting distant and occluded objects, which might not be recognized as an obstacle by typical CNNs. The presented tests show that the proposed technique enables a human to be detected at longer ranges, up to 90 m, preserving real-time operation.

In the literature, there are more approaches that use deep learning. In [24], the authors fine-tuned the popular AlexNet CNN [25]. The method was examined in terms of the ISO/DIS 18497 norm, which defines a barrel-shaped obstacle. To comply with the standard, the obstacle should be fully detectable by the system. The authors conclude that cameras may also be used as a part of safety systems, showing that precision is more than 90%.

Cameras are not an obligatory part of the obstacle detection system. Other studies show that only range sensors may be used. In [26], researchers utilize LIDAR data. The SVM algorithm based on the point cloud originating from sensors classifies points as obstacles, vegetation, or objects. The accuracy of the classification was 91.6%. Dvorak et al. [27] investigated the ultrasonic sensor in terms of the capability of detecting numerous obstacles and the possibility of finding in an agricultural field a human model, a Dracaena plant, a wooden fence, and more.

For more results on obstacle detection and techniques used in agricultural applications, the reader is advised to peruse the contents of the available survey articles [28,29,30].

### 2.2. Autonomous Lawn Mower

The article by Inoue et al. [31] investigates the operation of an autonomous lawn mower that is navigated without a global navigation satellite system (GNSS). Instead, they use cameras and IMU to simultaneously locate the mower, map the area (SLAM), and detect and avoid obstacles. This approach allows the mower to be used in areas where the GNSS signal is weak. The proposed approach was verified in a small 20 by 10 m lawn where the robot traveled a predefined path in the forward and backward directions. Using additional landmarks with known positions, the robot positioning accuracy at the end of the path is equal to 0.38 m and is worse when no landmarks are used. The authors report that an important factor of the error is the drift of the robot during turnaround. Results on obstacle detection and avoidance are not presented.

Visual-only obstacle detection for a lawn mower is presented by Franzius et al. [32]. As presented, the use of a low-cost camera and color-based obstacle detection can navigate the mower on a flat lawn, avoiding objects. In this approach, object detection is based on grass segmentation based on hue and saturation components of the image. The parameters of the segmentation are adapted based on the sequence of images to improve the accuracy of segmentation in variable lighting conditions. The robot operates under several assumptions regarding the operational area: the future driving path is on the same plane as the mower, obstacles lay on the ground, and the distance to the obstacle can be estimated from its pixel coordinates on the image. These assumptions eliminate the need for 3D information and simplify image processing and navigation but simultaneously restrict practical applications. As a result, good avoidance rates (above 90%) are achieved only for obstacles that do not stand out high from the ground. For poles, trees, and other plants, the avoidance rate is much smaller—unacceptable in industrial applications.

## 3. Obstacle Detection System Architecture

**Figure 1:**
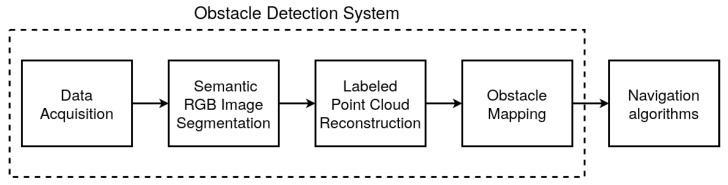
Obstacle detection system overview.

This section presents a novel RGB-D camera-based obstacle detection approach for mobile robots. The proposed method consists of four stages. First, the synchronized and aligned pair of an RGB image and a depth image is collected. Following this, the semantic mask of the environment is obtained based on the RGB image. Next, based on the semantic segmentation result and the depth image, the reconstruction of the point cloud occurs. Finally, the points are projected on the 2D occupancy grid [33], determining the position of obstacles on the map. The obtained grid is then utilized by the navigation algorithm. Figure 1 shows a general scheme of the proposed obstacle detection approach implemented using the robot operating system [34]. Each step of the system is described in more detail in the following subsections.

### 3.1. Autonomous Mower Platform Overview

The autonomous mower platform is a modified lawn mower for professional use, which is available on the market. Additional components were installed to reach the autonomous features, including sensors, actuators, and a computer. This provides a localization accuracy of 2 cm, which is a result of combining the Piksi Multi RTK module, SICK incremental encoders, and Xsens Mti-7 IMU data. Moreover, it is equipped with obstacle detection sensors which are four Intel RealSense D435i RGB-D cameras and two units of RP-Lidar-S1. All computations are done on an Intel Core i7 8700 PC equipped with an Nvidia GeForce 1050Ti and 16 GB of RAM. The mower can move at up to 0.4 m/s which gives a mowing efficiency at 1800 $m^{2}$/ha. Its width, depth, and height are equal to 1.64 m, 1.43 m, and 0.93 m accordingly.

The platform is equipped with a high-pressure hydraulic circuit that regulates and distributes the oil flow. In this application, the hydraulic system is powered by a gasoline engine that drives the hydraulic pump. The gasoline engine operates at a constant revolution rate to deliver constant power. The compressed oil flow is distributed by the hydraulic regulation circuit. In the analyzed scheme, two hydraulic motors are used to rotate the wheels, one for each side of the robotic platform. The wheel direction can be controlled by the solenoid valves, which are activated by binary signals. Depending on the valve configuration, the wheels can operate as follows: both sides in a forward direction, both sides in a backward direction, or each side in opposing directions. The last configuration is used to perform the skid steer maneuver, which allows the platform to execute a zero-radius turn [35].

### 3.2. Data Acquisition

**Figure 2:**
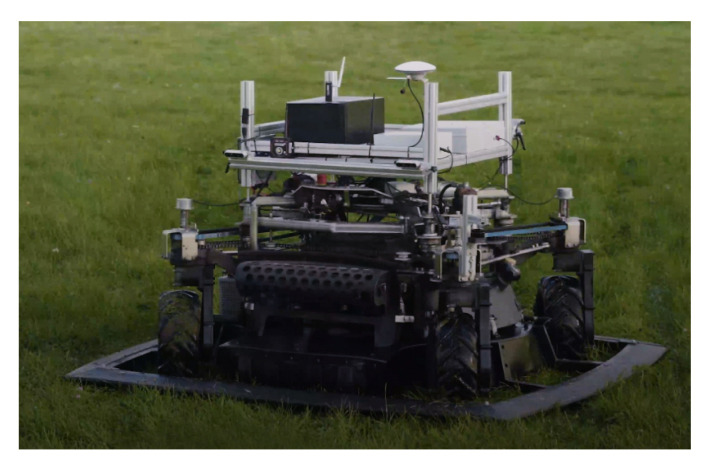
Autonomous mower platform used in the research.

The autonomous lawn mowing robot platform used in this study (Figure 2) has four Intel RealSense D435i [36] RGB-D cameras, one pair mounted at the front and the second one at the rear. Based on the mower’s direction of movement, only one pair of cameras is running.

**Figure 3:**
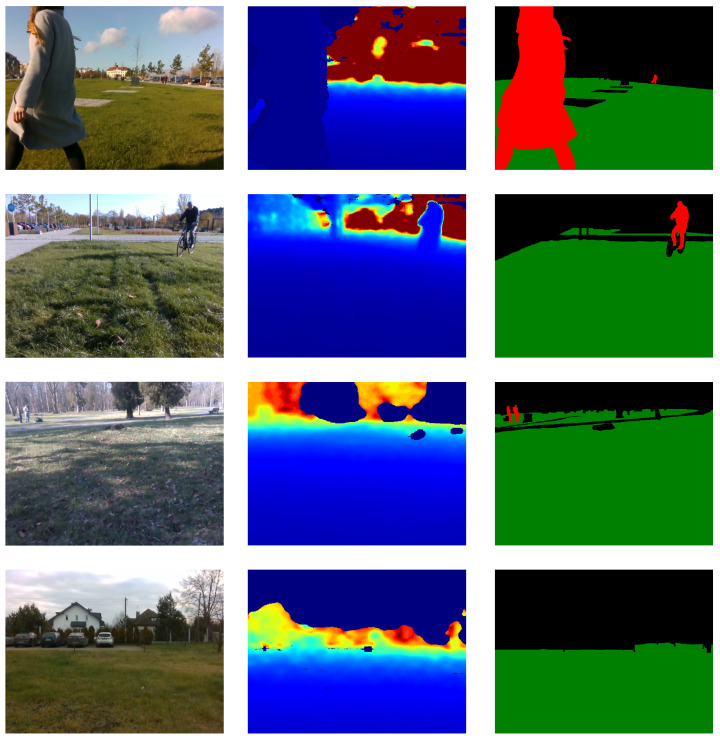
Examples of RGB images with corresponding depth maps and reference masks (from **left** to **right**) for each sequence: Boulevard 1, Boulevard 2, Park, and Backyard (from **top** to **bottom**).

The RealSense camera provides two types of images, RGB images and depth images. Examples of each are presented in the first and the second column, respectively, in Figure 3. The depth frame must be aligned with color camera coordinates. Alignment ensures that every pixel in the RGB image corresponds to exactly one pixel in the depth image. This property is then utilized in the labeled point cloud reconstruction step.

### 3.3. Semantic Image Segmentation

The goal of semantic segmentation is to classify each pixel in an RGB image into one of the given classes. This allows understanding the image on the pixel level. In the proposed approach, the set of predefined classes can be divided into two categories: one representing the working area of the robot and the other representing obstacles. In the case of autonomous lawn mowers [35,37], the class representing the working area is *grass*, and the example of the obstacle class is *person*. It is assumed that every pixel that is recognized as the *other* class is an obstacle. The list of classes can be adapted to different applications.

To solve the semantic segmentation task, two models were trained: the first one parses the scene into a *grass/obstacle* mask, and the second one recognizes grass, obstacles, and people. The chosen models’ architecture was the DeepLabv3+ [38] network with the pre-trained ResNet-34 [39] as the feature extractor, whereby the models vary in the last layer: the model *grass/obstacle* uses the Sigmoid activation function and the model *grass/obstacle/person* applies SoftMax over features to each spatial location. The segmentation results in a one-channel 448×448 mask image where pixel values represent the mower’s environment as follows: 0 implies class *other*, 1 indicates *grass*, and for the more complex model, the value 2 indicates the presence of the *person*. The mask is then up-sampled to match the depth image size. The models were trained using 52,502 pairs of RGB images and the corresponding image segmentation masks. The third column of Figure 3 shows the example results of semantic segmentation using the *grass/obstacle/person* model.

### 3.4. Labeled Point Cloud Reconstruction

**Figure 4:**
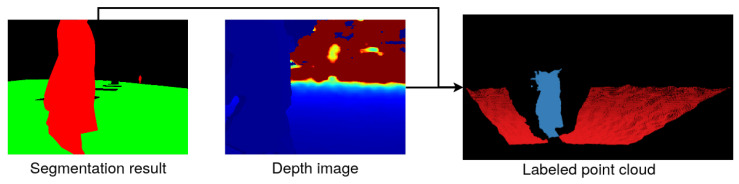
Example of the point cloud reconstruction—segmentation of the RGB image, depth image, and resulting point cloud with labels.

To obtain the labeled point cloud, the results of semantic segmentation and depth image are fused. This uses the depth to color alignment property described in Section 3.2. The point cloud consists of the XYZL points, which store the information about the coordinates of the point in the 3D world and contain the class label assigned during semantic segmentation. The point coordinates are computed as

(1) $$\left[\begin{array}{c} x\\ y\\ z \end{array}\right]=\frac{D(u,v)}{1000}K^{-1}\left[\begin{array}{c} u\\ v\\ 1 \end{array}\right],$$

where x,y,z are the real world coordinates, u,v are the image coordinates, D(u,v) is the depth value from the depth image in the point u,v, and *K* is the camera’s intrinsic matrix. The constant 1000 is applied to obtain the coordinates’ result in meters. An example of the point cloud reconstruction is shown in Figure 4. The resulting point cloud is then filtered and downsampled using the Point Cloud Library [40].

### 3.5. Obstacle Mapping

The final stage of the pipeline is the occupancy grid update. The occupancy grid (map) is used to represent a mobile robot workspace as a discrete grid [33]. The update procedure projects the [x,y] coordinates of points from the labeled point cloud onto the map cell’s coordinates $\left(x_{\mathrm{map}},y_{\mathrm{map}}\right)$:

(2) $$\left[\begin{array}{c} x_{\mathrm{map}}\\ y_{\mathrm{map}} \end{array}\right]=\left[\begin{array}{c} \left\lfloor \frac{x}{r}+x_{\mathrm{offset}}\right\rfloor \\ \left\lfloor \frac{y}{r}+y_{\mathrm{offset}}\right\rfloor \end{array}\right],$$

where $x_{\mathrm{offset}}$ and $x_{\mathrm{offset}}$ are offsets related to map origin and dimensions, and *r* is the map resolution in meters. The map update has two steps. The process starts with the map clearing of points which were classified as *grass*. Then, *obstacle/person* points are added to the map.

In order to include the result of the detection system in the navigation pipeline, the costmap layer, which is the occupancy grid representation in the ROS [34] library, was implemented using the framework proposed by Lu et al. [14].

## 4. Experiment Overview and Results

As was already mentioned in Section 3, our navigation module is highly dependent on the map, which is represented as an occupancy grid [33]. To finish mowing tasks successfully, reliable map updates are obligatory. The whole vision pipeline processing leads to adding or removing obstacles on the map. Therefore, the vision system evaluation should be considered from a map perspective. In the literature, numerous papers are available regarding obstacle detection systems for mobile robots including [15,17,32,41,42]. However, no article examines the proposed methods in terms of the generation of maps that contain information about working area, unknown places, and obstacle position.

In comparison with other evaluation methods which only test obstacle detection performance on images, i.e., [17,43], it has been noticed that our approach requires manual labeling of the point clouds, which is time consuming and may be affected by errors. However, such an approach allows obstacle detection systems to be compared in a 3D environment where navigation occurs. Moreover, our study has shown that image-based metrics are not sufficient to describe the whole obstacle detection system.

Our experiments could be divided into two separate categories. The goal of the first experiment was to investigate the image segmentation performance and calculate standard metrics for the classification problem, such as accuracy, F1, precision, recall, and specificity [44]. Then, based on point clouds, which were created from depth images, maps were generated. Maps may be considered as grayscale images and compared with reference maps using the same metrics as for the results of semantic segmentation. However, this evaluation method does not provide any information about the geometric properties of the errors, which are crucial to evaluate the safety of the navigation. For this reason, we propose a new metric $d_{\min-\mathrm{obst}}$ (minimum distance to obstacle), which is calculated for each point on the map that belongs to an FN (false negative) set, i.e., a point that is incorrectly classified as grass while it belongs to an obstacle. For a given FN point $p_{fn}$, the metric $d_{\min-\mathrm{obst}}$ is calculated as

(3) $$d_{\min-\mathrm{obst}}=\min_{tp_{i}}(∥\;p_{fn}-p_{tp_{i}}\;{∥}_{2}),$$

where the point $p_{tp_{i}}$ is the map cell correctly classified as the obstacle, TP is the set of all points correctly classified as obstacles, and $tp_{i}\in TP$. In the evaluation, we report the statistics of $d_{\min-\mathrm{obst}}$ values for all FN points on the map.

**Figure 5:**
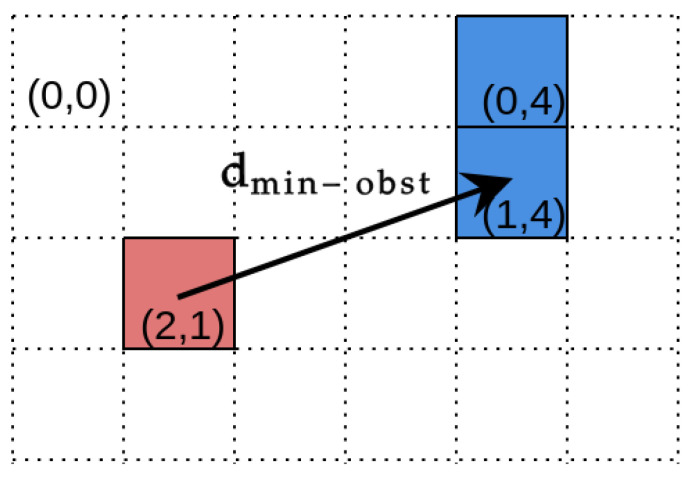
An example of the occupancy grid with a resolution of 1.0 m. The red color represents the map cells that were incorrectly marked as free space for robot operation (FN). Blue cells are the correctly detected obstacles (TP). The cell (2,1) contains an obstacle that was not detected, and the closest map cell to it that represents the obstacle is (1,4). Therefore, the $d_{\min-\mathrm{obst}}={∥\;\left(2,1\right)-\left(1,4\right)\;∥}_{2}\approx$ 3.16 m.

The idea behind the metric is straightforward. It calculates the Euclidean distance between the false negative map cell and the closest true positive. Figure 5 presents a graphical interpretation of the $d_{\min-\mathrm{obst}}$. The proposed metric can be used to estimate a margin for the robot to safely pass an obstacle.

In our research, we consider every pair of RGB and depth images as an input to the obstacle detection system that results in a standalone map. We use these maps to evaluate the quality of the proposed method. The results of the research are discussed in detail in Section 4.2 and Section 4.3.

### 4.1. Datasets Description

Our tests were conducted on two different datasets, i.e., synthetic and real world sets. The former is a multimodal, artificial dataset of Enclosed garDEN scenes (EDEN) [45]. The latter contains actual images and depth maps recorded by cameras mounted on the autonomous mower.

#### 4.1.1. Synthetic Dataset

We performed our tests using the subset of the EDEN (Enclosed garDEN) dataset, which contained images from 20 different synthetic gardens. For each scene, there are five sequences of images, which were generated in different lighting conditions. The dataset provides various modalities, but in this research, we used only RGB, depth, and semantic segmentation images. We used data obtained from the left camera. In total, 49,762 sets of data were used to evaluate the proposed obstacle detection system. The detailed structure of the test dataset is presented in Table 1.

#### 4.1.2. Real-World Dataset

**Figure 6:**
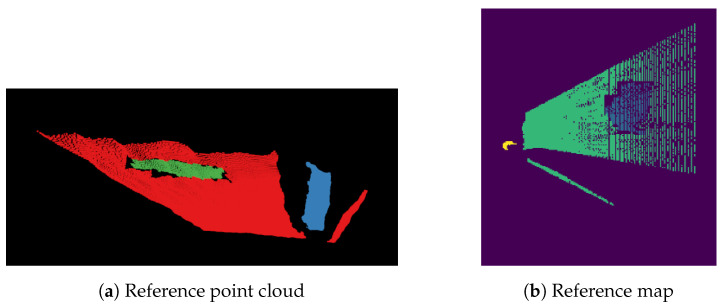
Example of (**a**) manually labeled point cloud (red—grass, green—obstacle, blue—person), and (**b**) reference map from Boulevard 1 sequence (green—grass, blue—obstacle, yellow—person, purple—unknown).

None of the synthetic datasets can be used to test the proposed solutions. Real-world application requires a realistic dataset which allows carrying out tests in diversified environments and in the presence of noise. Such an evaluation leads to conclusions about the advantages and disadvantages of the examined methods. This is why the second dataset was prepared. It consists of four sequences recorded at different places: boulevard, backyard, and park. An example of each sequence is shown in Figure 3. Each sequence contains two groups of data. The first group is dedicated to the image segmentation task. It has both RGB images and depth maps captured with Intel RealSense D435i camera with VGA resolution. Images were manually labeled using the Computer Vision Annotation Tool (CVAT), which is a free, open-source, browser-based application. It provides convenient annotation instruments [46]. The second group is intended to test obstacle detection on maps. For each sequence, five images were chosen, and based on these, the point clouds were generated. Then, reference point clouds were prepared using the RViz Cloud Annotation Tool [47]. Finally, reference maps were derived from the annotated point clouds. An example of a reference point cloud and reference map is shown in Figure 6.

### 4.2. Image Segmentation Results

The first stage of the research was to measure the performance of the two classification models on the images. The former model detects two classes, *other* and *grass*, while the more advanced one can also extract people. They were examined in terms of the following metrics: accuracy, F1, precision, recall, and specificity.

Table 2 and Table 3 contain results for the synthetic dataset and grass/obstacle model. In almost every illumination condition, the accuracy is higher than 98%. Only one exception can be observed for *sunset* lighting conditions, where the accuracy is slightly below 98%. The results presented in Table 2 indicate that different illumination conditions do not affect the classification performance significantly, which shows that our model is robust to lighting conditions. Regarding class-dependent metrics, the *grass/obstacle* model performs better for the *other* class in terms of F1, precision, and recall. These values are close to 99% for the *other* class and 91% for *grass*.

The results for the real-world dataset for both models are presented in the next two tables. Table 4 gathers calculated metrics for the *grass/obstacle* model, whereas Table 5 summarizes tests for *grass/obstacle/person*. The accuracy is higher than 96%. The only exception is the park sequence with 87.36%. This is the most challenging scene. The people are on the scene, but they are far away from the camera. Another difficulty is that the grass and footpath boundaries are not clear. This may explain the lower metrics in this case. However, the more advanced model copes with that sequence very well. Tests on real-life images collected on an autonomous mower in various lighting conditions have shown that we are able to correctly detect obstacles with high accuracy of 95.2%. This proves that our models are robust to harsh outdoor environments.

### 4.3. Map Generation Results

We consider the evaluation of the map generation process in two ways. In the first approach, we assume that the map is a grayscale image in which each pixel represents one of the classes. In the application of an autonomous lawn mower, these classes are grass, obstacle, person, and unknown. Depending on the model chosen for image segmentation, the map uses a proper subset of those classes. The label unknown indicates that the sensor has not covered this area. The quality of the generated map is obtained by comparison with the reference map and is described using the following metrics: accuracy, F1, precision, recall, and specificity. The second approach is more spatially oriented. It is used to analyze the distance from the map cell that is not recognized as an obstacle (although it contains an obstacle) to the closest point correctly recognized as obstacle using the $d_{\min-\mathrm{obst}}$ metric proposed in (3).

Table 6 and Table 7 show the results acquired for synthetic data. In the first, we can observe a significant performance loss of the classification executed directly on the maps compared to the results obtained for image segmentation (Table 3). This occurs for both resolution values; however, the maps generated in 0.05 m resolution are better at preserving information about objects’ locations. It can be also noticed that the recalls of the *grass* class, for both 0.05 m and 0.1 m resolutions, are considerably lower than other metrics. This observation reveals that map cells containing *grass* only are more likely to be misclassified as an obstacle. However, high *obstacle* class recall, above 97%, shows the robustness of the obstacle misclassification of the proposed obstacle detection system. This property is desirable from the safety system perspective. Despite the differences in classification metrics for the maps of various resolutions, the $d_{\min-\mathrm{obst}}$ measure shows that for resolutions of 0.05 m and 0.1 m, the system works with similar performance.

The *unknown* rows in Table 6 represent the map cells that have not been covered by sensors. Because this is an artificial dataset, there is no data noise, and for that reason, it is not possible to mark an unknown cell as an obstacle or grass.

In the case of the real world dataset, we need to deal with noise and distortion both in RGB and depth images. The results for the first approach performed on the data processed by the *grass/obstacle/person* model are presented in Table 8 and Table 9 for 10 cm and 5 cm resolution, respectively. For *Boulevard 1* and *Park* sequences, the map generation with the lower resolution (10 cm) has significantly better performance than the other one in terms of obstacle detection. This shows that the resolution parameter can function as a filter. However, in comparison with Table 5, significantly worse results were obtained. In particular, for the *Park* sequence, the precision of obstacle detection decreased from 92.5% to only 34.11% for 10 cm resolution and 26.13% for 5 cm. This proves that the obstacle detection system cannot be examined using RGB images only. It is also worth noting that the depth image noise is revealed by the metrics’ values for class *unknown*.

**Figure 7:**
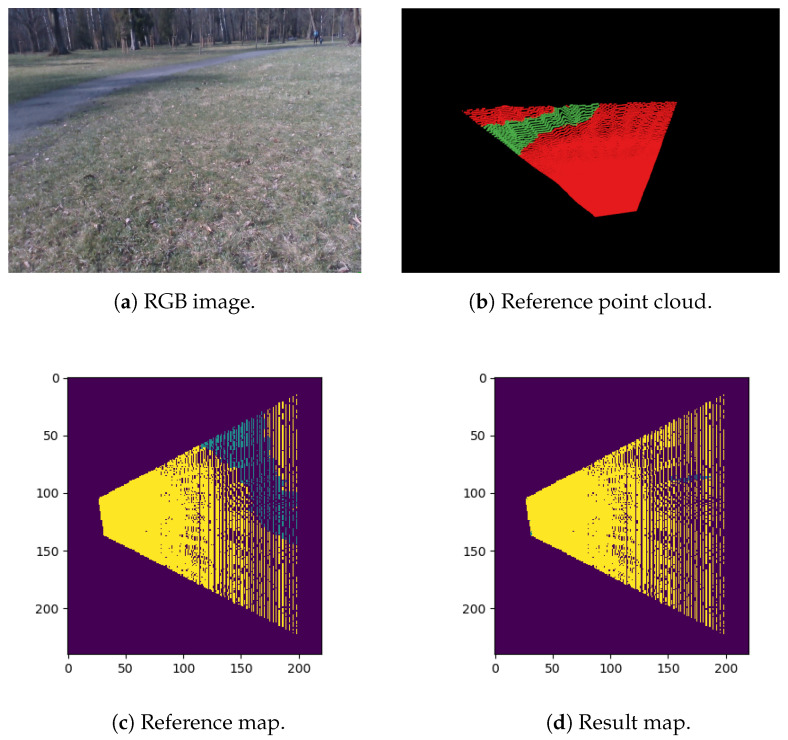
Example scene from the *Park* sequence: (**a**) presents the RGB image of the scene, (**b**) shows the labeled reference point cloud, and (**c**,**d**) are the reference map and the result of the obstacle detection pipeline, respectively. The maps’ resolution is 0.1 m, and the model *grass*/*obstacle*/*person* was used.

Table 10 contains the evaluation of the real world map results using the $d_{\min-\mathrm{obst}}$ metric. The analysis shows again that for the sequence *Park*, our system obtains the worst results. The average $d_{\min-\mathrm{obst}}$ for this sequence is 2.74 m and 3.13 m for a map resolution of 0.1 m and 0.05 m, respectively. However, this is the most challenging sequence from our dataset, and it appears that the model cannot detect the pathway in the scene as an obstacle correctly. The use of the $d_{\min-\mathrm{obst}}$ revealed the difficulty in detecting the obstacles in the *Park* scene which was not evident regarding the image-based evaluation. Figure 7 presents an example of a poorly detected dirt trail. We manage to get very good results for the sequence *Backyard*, but it is the most static scene from the real world dataset. Sequences *Boulevard 1* and *Boulevard 2* present dynamic scenes depicting humans. The first shows a woman walking right in front of the robot. The second one presents a biker stopping in front of the mower and riding away. The scene is more challenging because not only is the person moving, but the bike is also present, which represents the class *other*. For the second sequence, we achieved a mean value of the $d_{\min-\mathrm{obst}}$ equal to 0.38 m for the map resolution 0.1 m. This indicates the minimum value that should be added to the safety margin to navigate in a dynamic environment.

## 5. Conclusions

In this work, a methodology for accuracy determination in obstacle detection and mapping using RGB-D camera-based vision was proposed. In the proposed approach, artificial and real multimodal data containing a photo and 3D representation of the scene are used. These data are explored in the vision processing pipeline, where the first RGB picture is analyzed to detect obstacles in the working area. At this stage, it was shown that the scene segmentation obtains an average accuracy of 98.11%. Later on, the obstacles are matched with depth information for mapping the obstacle on the 3D map. Two data sources allowed the analysis to be performed in two parts, related to data processing issues (interpolation, resampling, point cloud size reduction, filtering, etc.) and hardware (sensor). It was determined that the average accuracy error introduced by the information processing is around 38 cm. The obtained value is considered in obstacle avoidance planning to establish the safety margin that is added to the detected obstacle radius. In this way, the safety margin can be established within real needs and not overestimated (the usual situation in practical implementations). Therefore, using the proposed approach in agricultural applications can lead to significant improvements in operational performance.

The proposed methodology can be used in other autonomous systems where the obstacle detecting system is based on RGB-D cameras. In this way, the safety margin can be adjusted to real requirements; as a consequence, more efficient path following and obstacle avoidance can be achieved. Finally, the task execution time can be reduced by limiting the robotic platform maneuvering, simultaneously keeping the required safety distance. The research also shows that obstacle detection is a complex system, and its performance cannot be assessed only based on image segmentation tests. The study proves that the final results of the obstacle detection system are affected by image segmentation, depth measurement errors, and point cloud reconstruction. Consequently, a comprehensive evaluation of the obstacle detection system needs to include navigation and mapping systems.

Future work will concentrate on increasing the system robustness in harsh, unknown environments, while maintaining long-term safety by using the $d_{\min-\mathrm{obst}}$ metric proposed in this paper. We also hope to look into the possibility of performing the scene segmentation step using both RGB and depth image data. Additionally, we are in the process of enhancing obstacle recognition performance for the group of obstacles that were identified in this research as challenging to recognize for the models used, such as dirt trails.

## Figures and Tables

**Table 1:** The synthetic dataset used for experiments contained the data from the images marked with the following numbers [45]: ‘0003’, ‘0005’, ‘0008’, ‘0018’, ‘0026’, ‘0029’, ‘0030’, ‘0040’, ‘0053’, ‘0068’, ‘0080’, ‘0160’, ‘0200’, ‘0214’, ‘0225’, ‘0257’, ‘0272’, ‘0313’, ‘0330’, ‘0347’.

Illumination Conditions	Clear	Cloudy	Overcast	Sunset	Twilight
Number of images sets	10,070	10,018	9519	10,055	10,100

**Table 2:** Segmentation results for *grass/obstacle* model under different illumination conditions.

Illumination Conditions	Class	Accuracy [%]	F1 [%]	Precision [%]	Recall [%]	Specificity [%]
Clear	Other	98.03	98.88	99.06	98.7	92.92
	Grass		91.67	90.45	92.92	98.7
Cloudy	Other	98.22	99.0	99.19	98.81	93.45
	Grass		92.0	90.58	93.45	98.81
Overcast	Other	98.13	98.94	99.15	98.73	93.54
	Grass		92.05	90.6	93.54	98.73
Sunset	Other	97.88	98.8	99.02	98.58	92.49
	Grass		90.9	89.37	92.49	98.58
Twilight	Other	98.04	98.9	99.07	98.72	92.63
	Grass		91.34	90.09	92.63	98.72

**Table 3:** Segmentation results for *grass/obstacle* model for synthetic dataset.

Class	Accuracy [%]	F1 [%]	Precision [%]	Recall [%]	Specificity [%]
Other	98.06	98.9	99.1	98.71	93.0
Grass		91.59	90.21	93.0	98.71

**Table 4:** Segmentation results for *grass/obstacle* model for real-world dataset.

Sequence	Class	Accuracy [%]	F1 [%]	Precision [%]	Recall [%]	Specificity [%]
Boulevard 1	Other	98.48	98.48	99.69	97.29	99.69
	Grass		98.49	97.32	99.69	97.29
Boulevard 2	Other	96.25	92.50	92.42	92.59	97.47
	Grass		97.50	97.53	97.47	92.59
Park	Other	87.36	75.06	73.19	77.02	90.75
	Grass		91.54	92.34	90.75	77.02
Backyard	Other	98.69	98.64	98.48	98.80	98.59
	Grass		98.73	98.88	98.59	98.80

**Table 5:** Segmentation results for *grass/obstacle/person* model for real-world dataset.

Sequence	Class	Accuracy [%]	F1 [%]	Precision [%]	Recall [%]	Specificity [%]
Boulevard 1	Other	98.22	98.06	97.07	99.07	97.52
	Grass	98.64	98.68	99.91	97.48	99.90
	Person	99.44	89.83	84.63	95.72	99.54
Boulevard 2	Other	96.28	92.41	92.33	92.49	97.51
	Grass	96.69	97.80	97.85	97.74	93.54
	Person	99.05	–	0.00	0.00	99.50
Park	Other	97.36	94.75	92.50	97.11	97.44
	Grass	97.50	98.32	99.12	97.55	97.34
	Person	99.83	22.32	25.42	19.89	99.93

**Table 6:** Obstacle detection results based on map updates using *grass/obstacle* model for synthetic dataset and various occupancy grid resolutions.

Map Resolution [m]	Class	Accuracy [%]	F1 [%]	Precision [%]	Recall [%]	Specificity [%]
0.05	Unknown	100.0	100.0	100.0	100.0	100.0
	Obstacle	99.45	94.5	92.06	97.08	99.57
	Grass	99.45	81.9	89.73	75.32	99.85
0.1	Unknown	100.0	100.0	100.0	100.0	100.0
	Obstacle	99.07	92.85	88.74	97.35	99.18
	Grass	99.07	72.62	88.25	61.69	99.83

**Table 7:** Summary statistics of the $d_{\min-\mathrm{obst}}$ measure evaluated on *grass/obstacle* model for synthetic dataset and various occupancy grid resolutions.

Map Resolution [m]	$\mathrm{avg}(d_{\min-\mathrm{obst}})$ [m]	$\mathrm{var}(d_{\min-\mathrm{obst}})$ [m]
0.05	0.16	0.02
0.1	0.17	0.03

**Table 8:** Obstacle detection results based on map updates using *grass/obstacle/person* model and 10 cm occupancy grid resolution.

Sequence	Class	Accuracy [%]	F1 [%]	Precision [%]	Recall [%]	Specificity [%]
Boulevard 1	Unknown	100.0	100.0	100.0	100.0	100.0
	Obstacle	100.0	100.0	100.0	100.0	100.0
	Grass	100.0	100.0	100.0	100.0	100.0
Boulevard 2	Unknown	99.33	99.52	99.06	100.0	97.75
	Obstacle	97.95	81.04	81.82	80.29	98.97
	Grass	98.33	96.51	97.66	95.39	99.27
Park	Unknown	99.08	99.31	99.31	99.31	98.60
	Obstacle	96.42	50.27	34.11	95.51	96.44
	Grass	95.69	92.69	98.38	87.63	99.34
Backyard	Unknown	100.0	100.0	100.0	100.0	100.0
	Obstacle	99.91	61.97	63.37	60.27	99.96
	Grass	99.99	99.87	99.92	99.92	99.96

**Table 9:** Obstacle detection results based on map updates using *grass/obstacle/person* model and 5 cm occupancy grid resolution.

Sequence	Class	Accuracy [%]	F1 [%]	Precision [%]	Recall [%]	Specificity [%]
Boulevard 1	Unknown	99.85	99.91	99.82	100.00	99.24
	Obstacle	98.74	62.02	99.42	45.06	99.99
	Grass	98.81	96.60	93.65	99.75	98.62
Boulevard 2	Unknown	99.60	99.75	99.50	99.99	97.93
	Obstacle	99.16	84.40	88.07	81.03	99.68
	Grass	99.34	98.00	98.52	97.49	99.71
Park	Unknown	98.73	99.19	99.22	99.16	97.20
	Obstacle	97.70	40.98	26.13	94.99	97.72
	Grass	96.73	91.73	97.05	86.96	99.30
Backyard	Unknown	100.0	100.0	100.0	100.0	100.0
	Obstacle	99.96	66.12	69.90	63.57	99.99
	Grass	99.97	99.92	99.97	99.96	99.98

**Table 10:** Summary statistics of the $d_{\min-\mathrm{obst}}$ measure evaluated on *grass/obstacle/person* model for real world dataset in different image sequences and 10 cm occupancy grid resolutions.

Sequence	Map Resolution [m]	$\mathrm{avg}(d_{\min-\mathrm{obst}})$ [m]	$\mathrm{var}(d_{\min-\mathrm{obst}})$ [m]
Boulevard 1	0.1	0.13	0.002
	0.05	0.1	0.003
Boulevard 2	0.1	0.38	0.18
	0.05	0.31	0.16
Park	0.1	2.74	5.5
	0.05	3.13	5.34
Backyard	0.1	0.14	0.01
	0.05	0.13	0.07

## Data Availability

EDEN dataset: https://lhoangan.github.io/eden/ (accessed on 3 August 2021).
